# Metabolic syndrome and the incidence of lung cancer: a meta-analysis of cohort studies

**DOI:** 10.1186/s13098-020-00598-0

**Published:** 2020-10-28

**Authors:** Li Qiao, Deliang Ma, Hui Lv, Ding Shi, Min Fei, Yu Chen, Fei Xie, Zhuoyan Wang, Ying Wang, Wanhua Liang, Peiying Hu

**Affiliations:** 1 Department of Oncology, Linyi Central Hospital, 17 Jian Kang Road, Linyi, 276400 Shandong China; 2 Health Promotion Center, Zhejiang Provincial People’s Hospital, People’s Hospital of Hangzhou Medical College, No. 158 Shangtang Road, Hangzhou, 310006 Zhejiang China; 3 State Key Laboratory for Diagnosis and Treatment of Infectious Diseases, College of Medicine, National Clinical Research Center for Infectious Diseases, Collaborative Innovation Center for Diagnosis and Treatment of Infectious Diseases, The First Affiliated Hospital, Zhejiang University, Hangzhou, 310003 Zhejiang China

**Keywords:** Metabolic syndrome, Lung cancer, Cohort study, Meta-analysis

## Abstract

**Background:**

Metabolic syndrome (MetS) has been related to the pathogenesis of variety categories of cancers. This meta-analysis aimed to determine the association between MetS and the incidence of lung cancer.

**Methods:**

Relevant cohort studies were identified by search of PubMed, Embase, and Cochrane’s Library databases. Cochrane’s Q test and I^2^ statistic were used to analyze the heterogeneity. Random-effect model which incorporates the potential heterogeneity was used for the meta-analysis.

**Results:**

Five cohort studies with 188,970 participants were included. A total of 1,295 lung cancer cases occurred during follow-up. Meta-analyses showed that neither MetS defined by the revised NCEP-ATP III criteria (hazard ratio [HR]: 0.94, 95% confidence interval [CI]: 0.84 to 1.05, p = 0.25; I^2^ = 0) nor the IDF criteria (HR: 0.82, 95% CI: 0.61 to 1.11, p = 0.20; I^2^ = 0) was associated with an affected risk of lung cancer. Subgroup analyses showed consistent results in women and in men, in studies performed in Asian and non-Asian countries, and in prospective and retrospective cohorts (p all > 0.05). Meta-analysis limited to studies with the adjustment of smoking status also showed similar results (HR: 0.91, 95% CI: 0.80 to 1.05, p = 0.21; I^2^ = 0). No publication bias was detected based on the Egger regression test (p = 0.32).

**Conclusions:**

Current evidence from cohort studies does not support that MetS is an independent risk factor for the incidence of lung cancer.

## Background

Metabolic syndrome (MetS) is a cluster of metabolic disorders characterized by the pathophysiological presence of central obesity, insulin resistance, high blood pressure, and dyslipidemia [1]. With the aging of the global population, MetS has become a common health problem in both the developed and the developing countries, with the reported prevalence of 10–30% of the adult populations [2–4]. Accumulating evidence confirmed that patients with MetS are at higher risk for the development of many other diseases, such as cardiovascular diseases [5], recurrent stroke [6], venous thromboembolism [7], sleep-disordered breathing [8], and osteoporosis [9].

Lung cancer is one of the most common cancers. In 2012, there were about 1.8 million new lung cancer cases and 1.6 million cases of death, respectively, accounting for about 13% of the total number of cancer diagnosis and 20% of the total number of cancer deaths [10]. Smoking is currently the most important risk factor for lung cancer, but there are still about 25% of lung cancer patients that are non-smokers [11]. Due to the high incidence and mortality related to lung cancer, identify risk factors for the pathogenesis of lung cancer is of important significance. Previous studies showed that MetS may be associated with cancer [12], probably due to their shared pathophysiological mechanisms such as low-grade chronic inflammation [13, 14]. However, subsequent studies showed that the association between MetS and cancer is likely to be site-specific the association between MetS and the incidence of lung cancer has not been fully determined [15]. In a previous meta-analysis [12], by including four cohort studies [16–19], the authors concluded that presence MetS did not affect the risk of lung cancer. However, besides studies reporting the incidence of lung cancer, they also included a study that reported the lung cancer mortality [16]. Since the outcome of cancer mortality could be affected by many clinical factors such as the anticancer treatments, including studies with mortality data may confound the overall result. Moreover, some subsequently published cohort studies were not included in the previous meta-analysis [20–22]. Therefore, we performed an updated meta-analysis to evaluate the association between MetS and subsequent incidence of lung cancer.

## Methods

We performed the meta-analysis in accordance with the MOOSE (Meta-analysis of Observational Studies in Epidemiology) [23] and Cochrane’s Handbook [24] guidelines.

### Literature search

Databases of PubMed, Embase and Cochrane’s Library were searched for relevant records. As for the search strategy, the combined terms were entered into the above databases as a single search, as ("metabolic syndrome" OR "insulin resistance syndrome" OR "syndrome X") AND ("lung" OR "pulmonary" OR "respiratory") AND ("cancer" OR "neoplasm" OR "carcinoma") AND ("prospective" OR "prospectively" OR "retrospective" OR "retrospectively" OR "followed" OR "follow-up" OR "cohort" OR "cohorts" OR "risk" OR "incidence"). We used this keywords search strategy instead of those searched as "text words" or as "Mesh terms" or “Emtree” to retrieve more comprehensive records. The search was limited to human studies published in English language. The reference lists of original and review articles were also analyzed using a manual approach. The final literature search was performed on April 20, 2020.

### Study selection

Articles were included in the meta-analysis if they met all the following criteria: (1) published as full-length article in English; (2) reported as cohort studies (prospective or retrospective, regardless of sample size) with the follow-up duration of at least one year; (3) included adult population (≥ 18 years of age) without lung cancer at baseline; (4) MetS defined according to the criteria of the original articles was identified as exposure of interest at baseline; (5) participants without MetS at baseline was considered as controls; (6) documented the incidences of lung cancer during follow-up; and (7) reported the adjusted hazard ratios (HRs, at least adjusted age and gender) and their corresponding 95% confidence intervals (CIs) for the incidence of lung cancer comparing individuals with MetS at baseline to those without MetS. Reviews, letters, editorials, preclinical studies and non-cohort studies were excluded.

### Data extracting and quality evaluation

Two authors independently performed literature searching, data extraction, and quality assessment according to the predefined inclusion criteria. Discrepancies were resolved by consensus. Data that were extracted include: (1) name of first author, year of publication and country where the study was performed; (2) design characteristics (prospective or retrospective); (3) characteristics and numbers of the participants; (4) criteria for the diagnosis of MetS; (5) follow-up period; (6) number of lung cancer cases in each study; and (7) variables adjusted when presenting the results. The quality of each study was evaluated using the Newcastle–Ottawa Scale [25] which ranges from 1 to 9 stars and judges each study regarding three aspects: selection of the study groups; the comparability of the groups; and the ascertainment of the outcome of interest.

### Statistical analyses

We used HRs as the general measure for the association between MetS at baseline and the incidence of lung cancer. Data of HRs and their corresponding stand errors (SEs) were calculated from 95% CIs or p values, and were logarithmically transformed to stabilize variance and normalized the distribution [24]. The Cochrane’s Q test and I^2^ test were used to evaluate the heterogeneity among the include cohort studies [26]. A significant heterogeneity was considered if I^2^ > 50%. We used a random-effect model to synthesize the HR data because this model is considered as a more generalized method which incorporates of the potential heterogeneity [24]. Sensitivity analyses, by removing individual study one at a time, were performed to test the robustness of the results [27]. Predefined subgroup analyses were performed to evaluate whether the association between MetS and lung cancer incidence was affected by gender of the participants, country of the study, and study design characteristics. Since smoking has been related with increased risk of lung cancer [28], we evaluated whether MetS is associated with lung cancer incidence in studies after adjustment of smoking. Moreover, potential publication bias was assessed by funnel plots with the Egger regression asymmetry test [29]. We used the RevMan (Version 5.1; Cochrane Collaboration, Oxford, UK) and STATA software for the meta-analysis and statistics.

## Results

### Literature searching

**Fig. 1:**
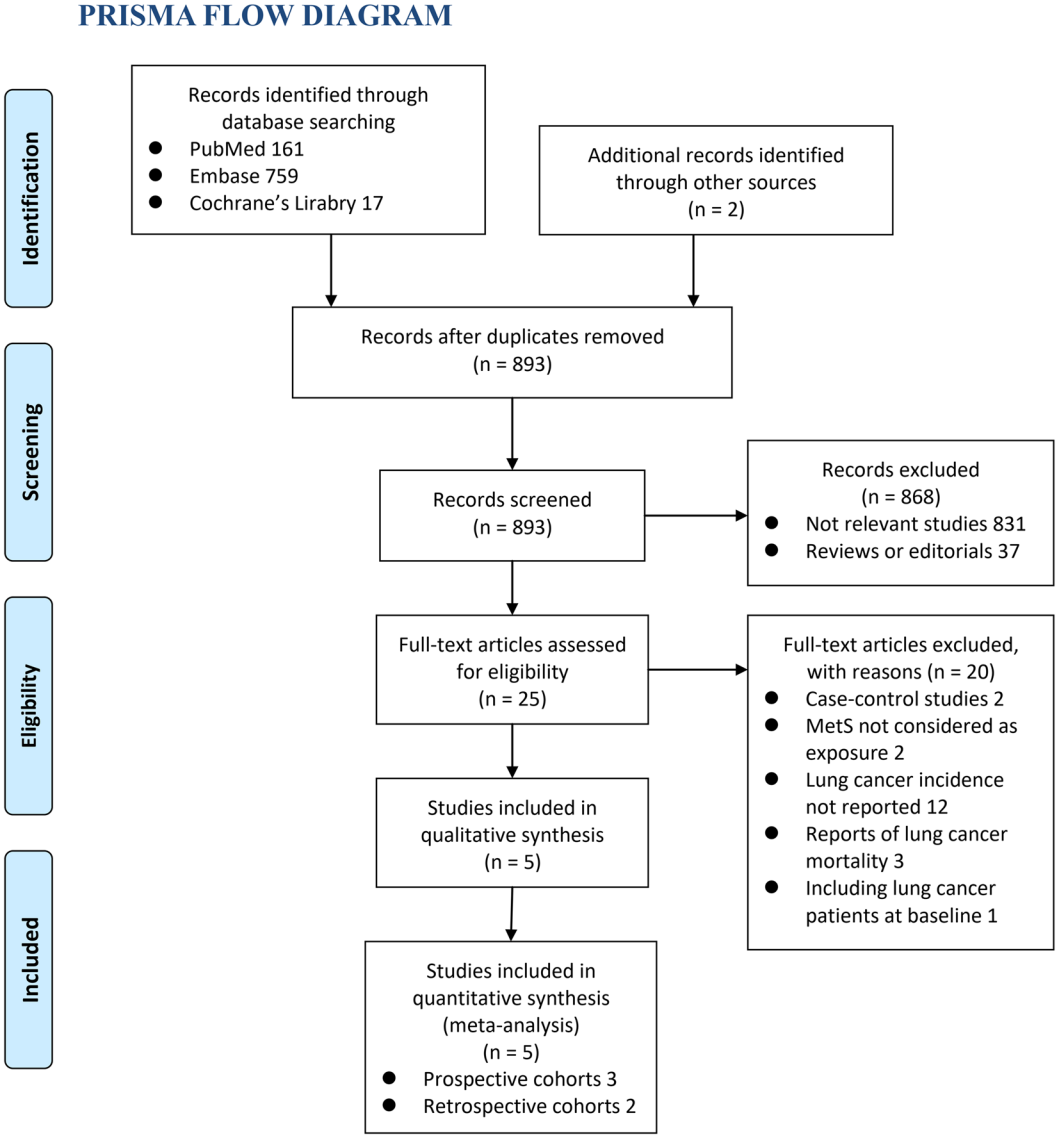
Flowchart for database search and literature screening

The processes of database searching were presented in Fig. 1. Briefly, 893 articles were found via initial literature searching of the PubMed and Embase databases, and 868 were excluded through screening of the titles and abstracts mainly because they were not relevant to the purpose of the meta-analysis. Subsequently, 25 potential relevant records underwent full-text review. Of these, 20 were further excluded for the reasons listed in Fig. 1. Finally, five cohort studies were included [17–21].

### Study characteristics and quality evaluation

The characteristics of the included cohort studies were presented in Table 1. Briefly, our meta-analysis included 188,970 participants from five cohorts. Two studies were performed in Europe [17, 20], and the other three were performed in Asia [18, 19, 21]. Regarding the design, two studies were retrospective [19, 21], whereas the other three were prospective [17, 18, 20]. Four of the studies included general populations [17–19, 21], whereas the other one included patients with vascular disease [20]. All of the included cohorts defined MetS according to the criteria of revised National Cholesterol Education Program’s Adults Treatment Panel III (NCEP-ATP III) [30], and two of them also included data of MetS diagnosed with the International Diabetes Federation (IDF) criteria [31]. Diagnosis of MetS according to NCEP ATP III or IDF criteria were based on the baseline measurements of the components of MetS combined with the confirmed treatments such as use of the antihypertensives or glucose-lowering agents among the included studies [17–21]. The incidence of lung cancer cases were mainly confirmed by the local cancer registries and 1,295 lung cancer cases occurred during follow-up. Age and gender were adjusted in all of the included studies when presenting the results, whereas smoking and alcohol intake were adjusted in four cohorts [18–21] except for one study [17]. The Newcastle–Ottawa scale varied from 7 to 9 in the included cohort studies (**Table ****2**), suggesting the generally good study quality.

**Table 1:** Characteristics of the included cohort studies

Study	Country	Design	Characteristics of the participants	Number of participants	Diagnostic criteria of MetS	Follow-up period	Diagnosis of lung cancer	Number of lung cancer cases	Outcome reported	Variables adjusted
Russo 2008	Italy	PC	Community based population	16,677	NCEP-ATP III	1999–2005	Local Cancer Registry	118	M, F, T	Age, gender
Inoue 2009	Japan	PC	Community based population	27,724	NCEP-ATP III and IDF	1990–2014	National cancer registries	224	M, F	Age, study area, smoking status, alcohol intake, daily total physical activity level, and TC
Osaki 2012	Japan	RC	General health examinees	38,832	NCEP-ATP III and IDF	1992–2007	Tottori prefectural cancer registry	211	M, F	Age, smoking status, alcohol intake
Kruijsdijk 2013	the Netherlands	PC	Patients with vascular diseases	6172	NCEP-ATP III	1996–2011	Netherlands Cancer Registry	118	T	Age, gender, smoking status, alcohol intake
Ko 2016	Korea	RC	National sample cohort for health check-up	99,565	NCEP-ATP III	2002–2013	Local Cancer Registry	624	M, F	Age, gender, smoking status, alcohol intake, and exercise

*NOS* the Newcastle–Ottawa Scale, *PC* prospective cohort, *RC* retrospective cohort, *MetS* metabolic syndrome, *NCEP-ATP III* National Cholesterol Education Program’s Adults Treatment Panel III, *IDF* International Diabetes Federation, *M* male, *F* female, *T* total, *TC* total cholesterol

**Table 2:** Quality evaluation of the included cohort studies via the NOS

Study	Representativeness of the exposed cohort	Selection of the non-exposed cohort	Ascertainment of exposure	Outcome not present at baseline	Control for age and sex	Control for other confounding factors	Assessment of outcome	Enough long follow-up duration	Adequacy of follow-up of cohorts	Total
Russo 2008	1	0	1	1	1	0	1	1	1	7
Inoue 2009	1	1	1	1	1	1	1	1	1	9
Osaki 2012	1	1	1	1	1	1	1	1	1	9
Kruijsdijk 2013	1	0	1	1	1	1	1	1	1	8
Ko 2016	1	1	1	1	1	1	1	1	1	9

*NOS* Newcastle–Ottawa Scale

### Association between the revised NCEP-ATP III defined MetS and lung cancer risk

**Fig. 2:**
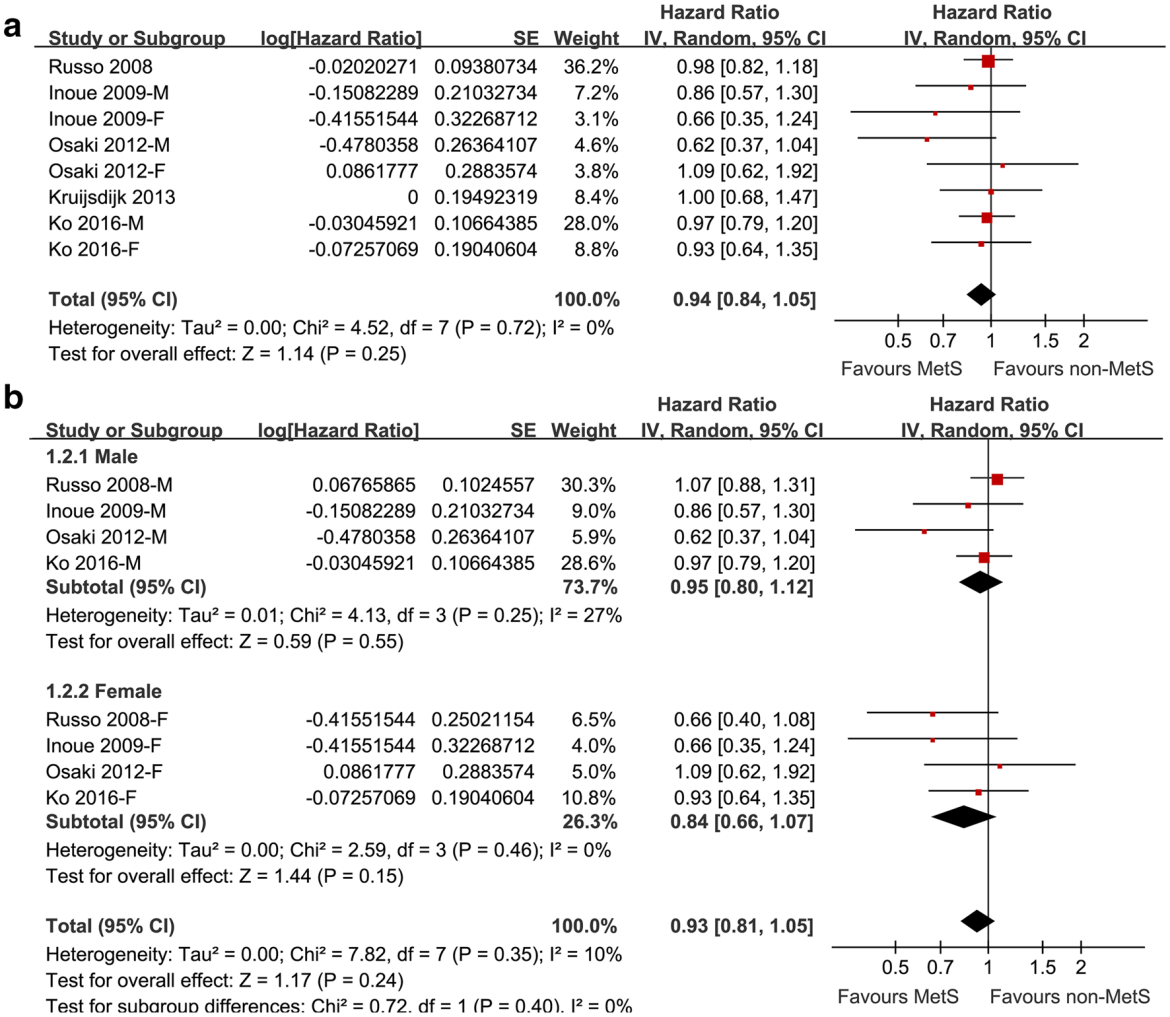
Forest plots for the meta-analysis of the association between the revised NCEP-ATP III defined MetS and lung cancer risk. **a** forest plots for the overall participants; **b** forest plots for the subgroup analysis by gender

**Fig. 3:**
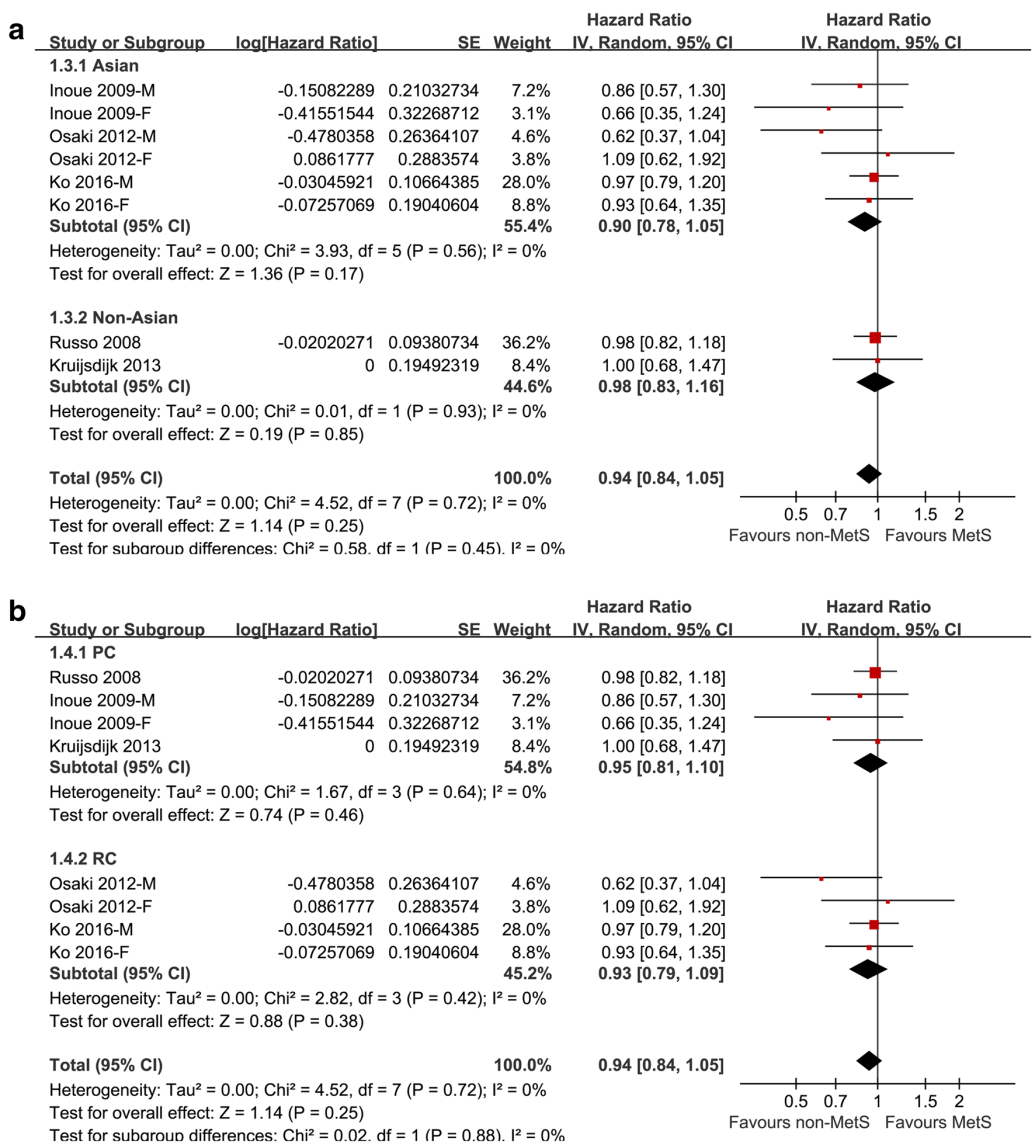
Forest plots for the subgroup analysis of the association between the revised NCEP-ATP III defined MetS and lung cancer risk. **a** forest plots for the subgroup analysis by study country; **b** forest plots for the subgroup analysis by study design

Five cohort studies [17–21] with 188,970 participants reported the association between MetS diagnosed by revised NCEP-ATP III at baseline and the subsequent risk of lung cancer incidence. Since three of them [18, 19, 21] reported the outcomes separately according to the gender of the participants, a total of eight datasets were included. Result of the meta-analysis did not support a significant association between MetS and the risk of lung cancer (adjusted HR: 0.94, 95% CI: 0.84 to 1.05, p = 0.25; Fig. 2a) with no significant heterogeneity (p for Cochrane’s Q test = 0.72, I^2^ = 0). Results of sensitivity analyses by excluding one study at a time did not significantly affect the result, suggesting the stability of the main result (Table 3). Specifically, excluding the study [17] in which smoking status was not adjusted showed similar result (adjusted HR: 0.91, 95% CI: 0.80 to 1.05, p = 0.21) with no significant heterogeneity (p for Cochrane’s Q test = 0.65, I^2^ = 0). Results of subgroup analyses according to the gender of the participants were also similar (for male: adjusted HR: 0.95, 95% CI: 0.80 to 1.12, p = 0.55, I^2^ = 27%; for female: adjusted HR: 0.84, 95% CI: 0.66 to 1.07, p = 0.15, I^2^ = 0; Fig. 2b). The difference between the results in male and female participants was not statistically significant (p = 0.40; Fig. 2b). In addition, subgroup analyses showed similar results in studies performed in Asian and non-Asian countries (p > 0.05, Fig. 3a), and in prospective and retrospective cohort studies (p > 0.05, Fig. 3b).

**Table 3:** Results of sensitivity analysis

Studies omitted	HR	95% CI	I^2^	P for effect
Russo 2008	0.91	0.80 to 1.05	0	0.21
Inoue 2009-M	0.94	0.84 to1.06	0	0.32
Inoue 2009-F	0.95	0.85 to1.06	0	0.35
Osaki 2012-M	0.96	0.85 to1.07	0	0.44
Osaki 2012-F	0.93	0.83 to1.04	0	0.22
Kruijsdijk 2013	0.95	0.83 to1.04	0	0.23
Ko 2016-M	0.93	0.81 to1.05	0	0.24
Ko 2016-F	0.94	0.84 to 1.65	0	0.28

*HR* hazard ratio, *CI* confidence interval

### Association between IDF defined MetS and lung cancer risk

**Fig. 4:**
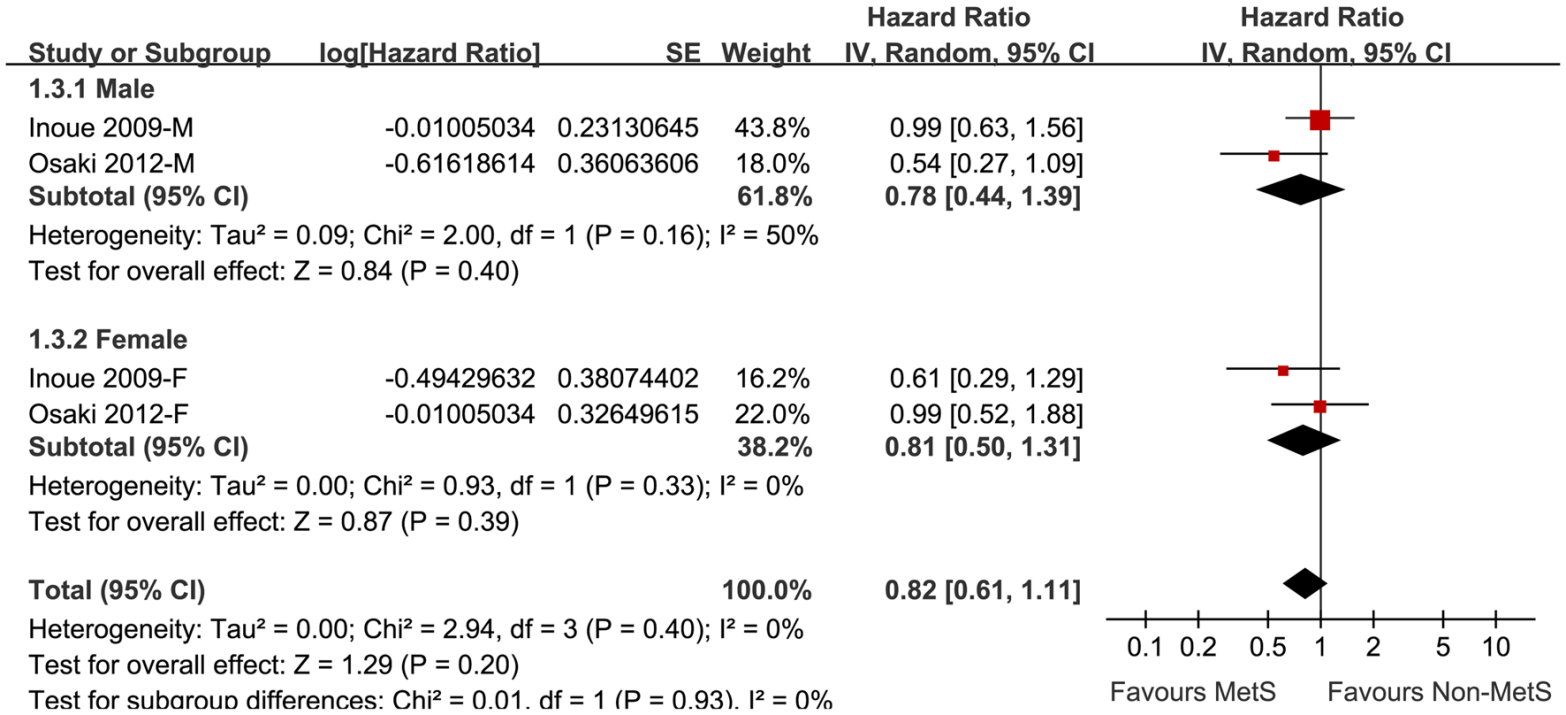
Forest plots for the meta-analysis of the association between IDF defined MetS and lung cancer risk stratified by gender;

Two cohorts [18, 19] with 66,556 participants reported the association between IDF defined MetS and the subsequent risk of lung cancer. Results of the meta-analysis did not show a significant association (adjusted HR: 0.82, 95% CI: 0.61 to 1.11, p = 0.20; I I^2^ = 0; Fig. 4). Results of subgroup analyses according to the gender of the participants were also similar (for male: adjusted HR: 0.78, 95% CI: 0.44 to 1.39, p = 0.40, I^2^ = 50%; for female: adjusted HR: 0.81, 95% CI: 0.50 to 1.31, p = 0.39, I^2^ = 0; Fig. 4). The difference between subgroups was not statistically significant (p = 0.93).

**Fig. 5:**
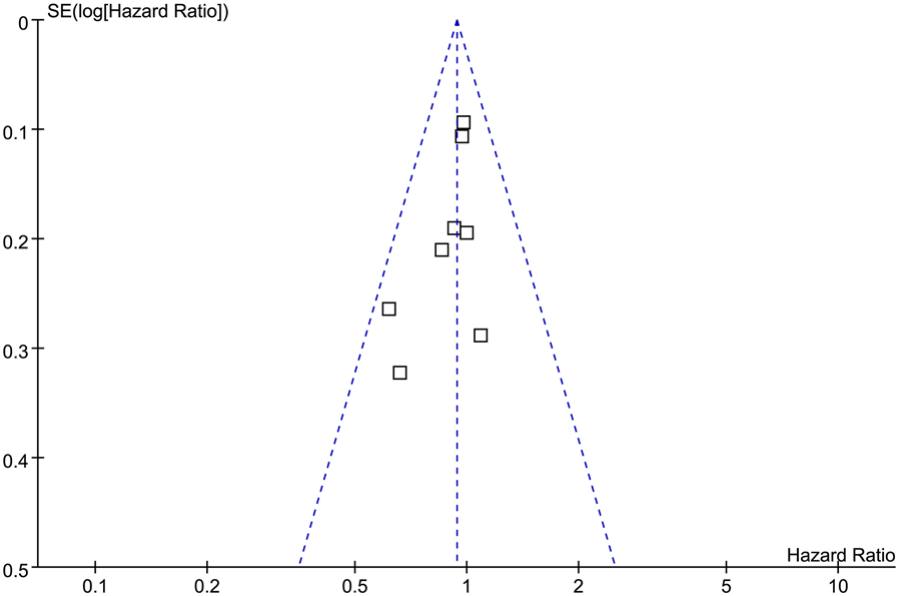
Funnel plots for the meta-analysis of the association between the revised NCEP-ATP III defined MetS and lung cancer risk

### Publication bias

The funnel plot regarding MetS diagnosed by revised NCEP-ATP III at baseline and risk of cognitive decline was shown in Fig. 5. The funnel plot was symmetry on visual inspection. Results of Egger regression test suggested that no significant publication bias was detected (p = 0.32). The publication bias for the meta-analysis of association between IDF defined MetS and the subsequent risk of lung cancer was difficult to estimate since limited cohorts were included.

## Discussion

In this meta-analysis, by pooling the results of five cohort studies of 188,970 participants, the result showed that presence of MetS does not significantly influence the subsequent incidence of lung cancer. The results were consistent in male and female participants, in studies performed in Asian and non-Asian countries, in prospective and retrospective cohorts, and in studies in which smoking habit was adjusted in multivariate analyses. Moreover, the association between MetS and lung cancer incidence was not affected by the definitions of MetS (based on the revised NCEP-ATP III or IDF criteria). These results suggested that current evidence from cohort studies does not support that MetS is an independent risk factor for the incidence of lung cancer.

Results of our meta-analysis may reflect the fact that the components of MetS may have different influences on the risk of lung cancer. For the association between obesity and lung cancer risk, current evidence is not consistent. A previous meta-analysis 31 studies showed that obesity is protective factor against lung cancer [32], whereas another study in Chinese patients suggested that the protective effect of obesity against lung cancer may be confounded by the smoking status [33]. Subsequent meta-analysis of six prospective cohort studies indicated that abdominal obesity may be a risk factor for the incidence of lung cancer [34]. Interestingly, a recent meta-analysis with 28 prospective cohort studies suggested a significantly positive relationship between waist circumference, rather than BMI, and lung cancer risk, suggesting there might have an etiological connection between central obesity and lung cancer development [35], rather than overall obesity as evidenced by increased BMI. Moreover, as for the lipids profiles, recent evidence indicated that higher high-density lipoprotein cholesterol level is protective against the lung cancer, whereas higher triglyceride is associated with higher lung cancer incidence [36], and these findings are confirmed by a subsequent case–control study in Chinese patients with and without non-small cell lung cancer [37]. However, a recent prospective cohort study showed that the association between triglyceride and lung cancer risk may be more complicated than expected and presented as a U-shaped association [38]. In addition, results regarding the association between hypertension and lung cancer risk are inconsistent. The result Metabolic Syndrome and Cancer Project indicated a small increased lung cancer risk in men with elevated blood pressure level, but not in women [39]. However, an early study in Korean men showed that hypertension was not an independent risk factor in lung cancer mortality [40]. Similarly, result of a meta-analysis of 14 cohort studies also concluded that diabetes was not associated with lung cancer risk [41]. A recent cohort study showed that the potential impact of diabetes on the risk of lung cancer may be modified by smoking status of the patients, and diabetes may have minimal impact on lung cancer development in the never-smoking population [42]. Taken together, it seems that association between the components of MetS and the risk of lung cancer remain uncertain and may be modified by many factors including smoking status. Additionally, since people with MetS often have unhealthy life styles, such as smoking, alcohol drinking and less exercise, these factors may also confound the association between MetS and lung cancer. Most of the included studies in our meta-analysis have adjusted these factors, which may therefore weaken the association between MetS and Lung cancer incidence. Moreover, accumulating evidence showed that treatments against the components of Mets, such as the use of metformin, may lead to a reduced risk of lung cancer incidence [43]. Whether these factors may confound the association between MetS and Lung cancer risk also deserves further investigation.

The strengths of our study, compared with the previous meta-analysis [12] may include the followings. Firstly, we included only studies with multivariate analyses, which minimized the potential influences of study or participant characteristics on the outcome. Secondly, only studies reporting the incidence of lung cancer were included, rather than the studies that reported the morality of lung cancer. Since lung cancer mortality could also be affected by treatment status after diagnosis, the previous meta-analysis combining the data of lung cancer incidence and mortality may confound the results [12]. Finally, we included five cohort studies with eight datasets, which enabled us to perform multiple stratified analyses to confirm the findings of the main analysis. Despite of this significance, our study also has limitations which should be considered when interpreting the results. Firstly, as a meta-analysis of observational studies, results of our study did not support a sequential association between MetS and lung cancer incidence. Since lung cancers of different histopathological type may have different biological features, the association between MetS and different histopathological type of lung cancer should be analyzed. However, since data according to the histopathological type of lung cancer were not reported in either of the included cohort studies, we were unable to evaluate the outcomes according to the histopathological type of lung cancer. Future studies are warranted in this regard. Secondly, although MetS defined by revised NCEP-ATP III or IDF criteria was not associated with lung cancer incidence, association between MetS defined by other criteria and subsequent lung cancer incidence remains undetermined. Thirdly, although our study combined the data of 188,970 participants and 1295 cases of lung cancer, we could not fully exclude the possibility that the meta-analysis is statistically underpowered for the detection of the association between MetS and lung cancer incidence. Finally, as previous mentioned, although we combine multivariate adjusted data, we could not exclude that there remains residual factors that may confound the result, such as smoking status, dietary factors, and concurrent medications including metformin et al.

In conclusion, results of our meta-analysis showed that current evidence from cohort studies does not support that MetS is an independent risk factor for the incidence of lung cancer. The influences of each component of MetS on pathogenesis of lung cancer should be evaluated in future studies.

## Data Availability

The available data and materials section refers to the raw data used in our study are included in manuscript with tables, figures and its supplementary information files. All the authors agreed that the data could be shared if researchers required.

## Abbreviations

MetS: Metabolic syndrome
MOOSE: Meta-analysis of Observational Studies in Epidemiology
HRs: Hazard ratios
SE: Stand error
NCEP-ATP III: Revised National Cholesterol Education Program’s Adults Treatment Panel III
IDF: International Diabetes Federation
